# Ground air pollutants explanation based on multiple visibility graph of complex network by temporal community division

**DOI:** 10.1371/journal.pone.0291460

**Published:** 2024-03-07

**Authors:** Chubing Guo, Jian Wang, Yongping Zhang, Haozhe Zhang, Haochun Yang

**Affiliations:** 1 Xidian University, School of Artificial Intelligence, Xi’an, Shaanxi, China; 2 CETC Key Laboratory of Data Link Technology, Xi’an, Shaanxi, China; 3 AVIC Chengdu Aircraft Design & Research Institute, Chengdu, Sichuan, China; 4 School of Computer Science and Engineering, Northwestern Polytechnical University, Xi’an, Shaanxi, China; National Cheng Kung University, TAIWAN

## Abstract

In air pollution studies, the correlation analysis of environmental variables has usually been challenged by parametric diversity. Such variable variations are not only from the extrinsic meteorological conditions and industrial activities but also from the interactive influences between the multiple parameters. A promising solution has been motivated by the recent development of visibility graph (VG) on multi-variable data analysis, especially for the characterization of pollutants’ correlation in the temporal domain, the multiple visibility graph (MVG) for nonlinear multivariate time series analysis has been verified effectively in different realistic scenarios. To comprehensively study the correlation between pollutant data and season, in this work, we propose a multi-layer complex network with a community division strategy based on the joint analysis of the atmospheric pollutants. Compared to the single-layer-based complex networks, our proposed method can integrate multiple different atmospheric pollutants for analysis, and combine them with multivariate time series data to obtain higher temporary community division for ground air pollutants interpretation. Substantial experiments have shown that this method effectively utilizes air pollution data from multiple representative indicators. By mining community information in the data, it successfully achieves reasonable and strong interpretive analysis of air pollution data.

## 1. Introduction

Ground air pollution is currently one of the most severe and worrisome environmental problems in China. Among the air pollutants, PM_2.5_, O_3_, and SO_2_ are the main pollution contaminants and the major indexes for pollution measurement as well [1]. Especially for the PM_2.5_ particle, it contains a large number of toxic and harmful substances in small size and is able to suspend in the atmosphere for a long time during long-distance transportation. The air pollutants cause people to suffer from lung diseases, cardiovascular diseases, and so on, which also leads to a motivation of worldwide related research [2, 3]. Similar to PM_2.5_, SO_2_ also induces various respiratory diseases. Moreover, SO_2_ in the air has the capability to help the formation of suspending particles, which enhance the concentration of PM_2.5_, PM_10_, and other inhalable particles. For another pollutant O_3_, the study of Doherty and Miao showed that this type of photochemical oxidant had a critical impact on air quality due to its abundance, and its potential negative impact on global economic losses would even reach billions of dollars [4, 5]. In China, the intensively increased O_3_ has also become a crucial factor for air quality decline in recent years.

Relevant research [6, 7] has demonstrated that the formation and diffusion of the above pollutants can be influenced nonlinearly by various factors. Besides temperature, other natural meteorological conditions such as precipitation, humidity, and even the development of the economy can also become the distinctive factors to impair the air quality by taking the emitted gases and fossil fuels of industrial production into consideration. Particularly, some of those pollutants discharged into the atmosphere would also form secondary pollution via photochemical reaction, which indicates an inherent interaction between pollutant factors.

In more recent studies, analysis of time series data based on the visibility graph (VG) has obtained significant progress [8]. Yu et. al. employed a finite visibility graph to analyze the dynamic information change of sea surface temperature (SST) [9]. Based on a single-layer complex network, Yu’s model reveals a strong correlation between the SST variability and El Niño events. In a similar way, Cabezas et.al utilized a VG model to obtain the relevant parameters of tropospheric O_3_ data [10], and further explore the environmental distinction between urban and rural areas. Whereas the complex network has been applied in different applications for time series data analysis, a more comprehensive understanding of multiple indexes with correlation in the same time period is still limited due to the disconnection of single/multiple indexes. Additionally, since the single index analysis does not obey the objective and cognitive laws of the mutual influence between different objects, an incomplete description of the information is hard to be avoided either.

Multivariate time series refers to a type of data where multiple variables are observed and recorded over time. In a time series, data points are collected sequentially at regular intervals, such as daily, weekly, or monthly, and each data point corresponds to a specific time stamp. Differently, Nicosia et.al. proposed a mechanism to transform multivariable time series into multiple visualizations [11], which was also employed by Stephen et. al. [12] to perform dynamic phase detection and community detection on pedestrian data. In [12], the pedestrian trajectory data were converted into multiple networks based on the theory of complex networks and found that the simple indicators of multiple visualizations are able to accurately represent the global dynamic stages and local communities within the scene.

All of these studies have shown that more comprehensive information can be effectively characterized by multi-dimensional data analysis to benefit the inherent data correlation, especially for the clustering problems such as community division. To achieve more effective multi-dimensional data analysis, in this work, a joint modeling strategy is proposed by incorporating the MVG with temporal community division. The proposed solution is initialized by converting the pollution data with multiple indicators into a multi-complex network using MVG, which is to perform an analysis of its intrinsic complex network indicators. Then, the communities’ information of different sizes obtained from multiple networks is categorized using community detection for further investigation. With more accurate community division realization, the correlation between pollutant data and time can be described more stably, especially when compared to the other viewable schemes based on single-layer networks.

## 2. Materials and methods

### 2.1 Visibility graph

A network can be represented as a graph G containing point set V and edge set E. By transforming the time series information for complex network representation [13, 14], this graph is named visibility graph (VG) which is able to carry sufficient properties of the original time series signal [8]. Usually, the visibility graph employs a visibility matrix to store the information of all nodes in the graph. When the matrix is generated, the most important process is to determine the condition of judging whether the two points are visible to each other according to the criterion that:

Suppose two arbitrary data from the time series (*t*_*a*_, *y*_*a*_) and (*t*_*b*_, *y*_*b*_) are visible, the two corresponding nodes are connected to each other in the visibility graph, only if any other data point (*t*_*c*_, *y*_*c*_) between them (*t*_*a*_<*t*_*c*_<*t*_*b*_) fulfills the following condition:

$$y_{c}<y_{a}+(y_{b}-y_{a})\frac{t_{c}-t_{a}}{t_{b}-t_{a}}$$ (1)

By applying visibility graph, an N*N adjacency binary matrix can be obtained, in which each element carries the information about the corresponding node. E.g. an element *a*_*ij*_ = 1 from row *i* and column *j* represents that the node *i* and node *j* are visible accordingly. Meanwhile, the element value in row *j* and column *i* is the same *a*_*ji*_ = 1 because of the symmetry property of the resulting matrix.

**Fig 1:**
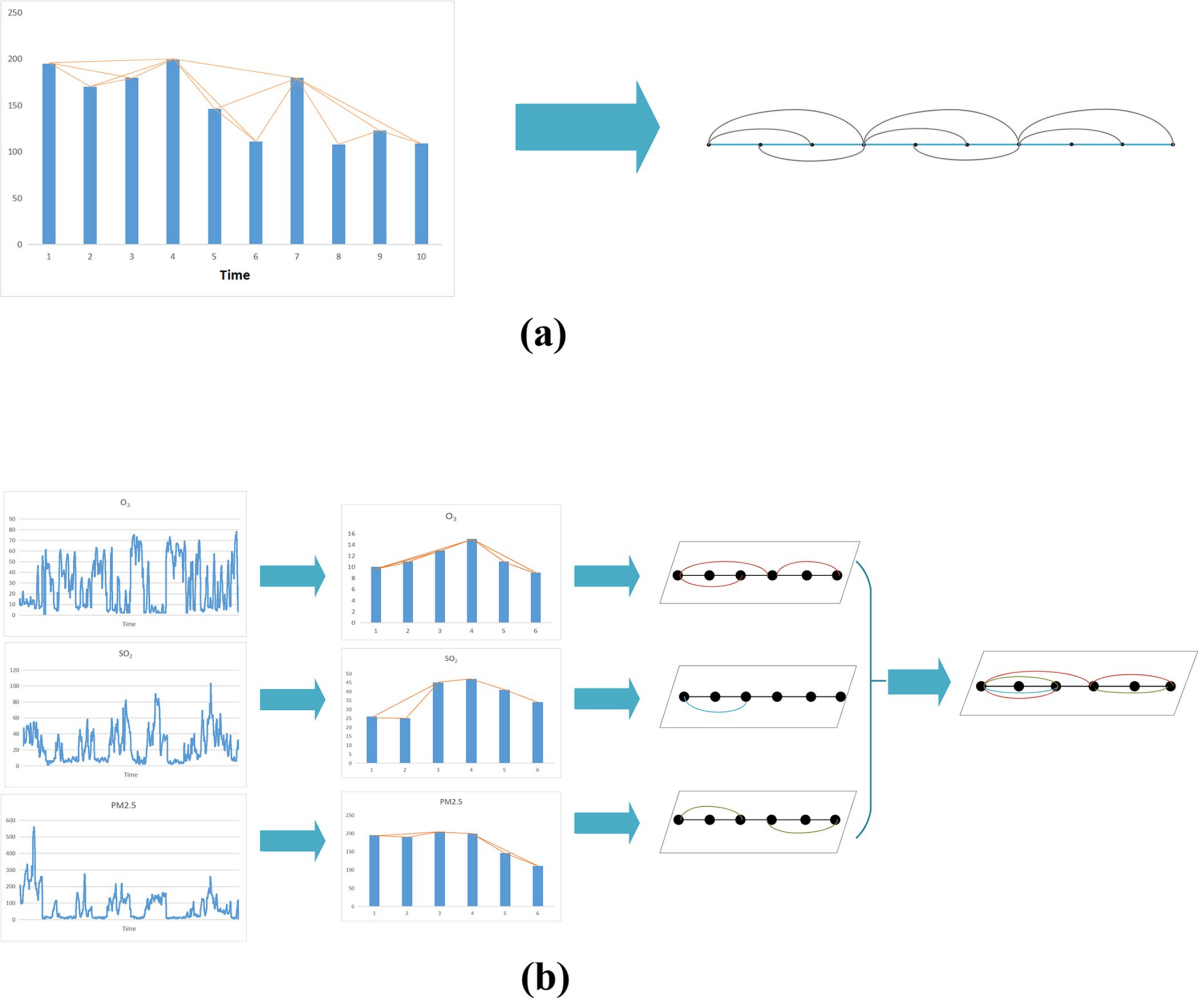
Schematic diagram of converting multiple time series into Multiplex Visibility Graph (a) The visibility graph and its associated graph; (b) The procedure to construct the multi-layer time series networks.

Fig 1(A) shows the visibility graph-based transformation from the time series data to complex networks. Given the time series data in the left, the generated topology of the complex network is in the right. The orange line in the left graph indicates that the node has a connection relationship, which is also reserved as a straight line or curve for node connection in the right graph.

### 2.2 Multiplex Visibility Graph

The Multiplex Visibility Graph (MVG) is a graph-based representation and analysis method used for studying multivariate time series data. It is an extension of the visibility graph approach, which is a technique for transforming time series into graphs to uncover patterns and relationships. For the data of multivariate time series, which is composed of M indicators, a multi-layer network [9] called multiple visibility graph (MVG) can be used to describe the VG of a single variable in the multivariate time series corresponding to each layer [15], as shown in Fig 1(B).

### 2.3 Degree centrality

To specifically describe the statistical properties of complex networks related to the characteristic attributes of networks, centrality parameters are introduced as a convenient mathematical tool [16, 17]. Considering that the degree distribution is a central parameter commonly used in complex networks, in this work, the degree of a point is defined as the number of nodes visible to it, which is the number of nodes connected to the node in the graph as well. In different networks, the meaning of degree may not be the same, e.g. in a social network, the greater the degree of the individual is, the greater impact one would have, and vice versa.

The degree distribution can be represented by a probabilistic function of node degree *P*(*k*) which is used to calculate the nodes’ probabilities of different degrees in the network. For time series analysis, more hidden information can be obtained based on this distribution [13, 18, 19]. When a visibility graph is associated with a fractal time series, the right tail of its degree distribution function can be fitted by a power function as *P*(*k*)∝*k*^−*γ*^. In a log-log plot, an index coefficient γ can be obtained by fitting *P*(*k*) into a linear regression curve, and show an influential capability on the dynamic properties of the network.

### 2.4 Community detection

The common feature of complex networks is that they are generally structured by communities. As a subgraph of a complex network diagram, the defined property of the community is that nodes inside the community have stronger connections than those outsides. In recent years, mining the significance of community structure has attracted an increased number of studies on different types of large-scale complex networks, such as Kernighan-Lin [20], GN [21], Spectral Average [22] and so on [23, 24]. To further evaluate the mining performance of the obtained community structure, the metric of modularity has been adopted based on the modular degree in complex networks from 2003 [25]. Later in [26], the definition of this metric was updated to the fraction of edges falling in the given group minus the probability fraction obtained by randomly distributing these edges. Suppose that the network has *n* nodes and *m* edges, the degree of node *v* is expressed as *k*_*v*_, the adjacency matrix of the network is *A*_*nn*_, where *A*_*vw*_ = 0 indicates that there are no edges between node *v* and node *w*, while *A*_*vw*_ = 1 indicates that the pair nodes are connected by the elaborative explanations as below:

1. *s*_*vw*_ = 1 means that node *v* and node *w* belong to the same community, while *s*_*vw*_ = 0 does not. The formula *δ*_*vw*_ = (*s*_*vw*_+1)/2 can be quantified to decide whether *v* and *w* are in the same community. If yes, *δ*_*vw*_ = 1; otherwise *δ*_*vw*_ = 0. Then, the ratio of edges belonging to the same community of all the edges can be calculated by:

$$\left(\sum_{vw}\left(A_{vw}∙\delta_{vw}\right)\right)/2m$$ (2)

1. In the random networks, the expectation of the edges number connecting node v and node w is $\frac{k_{v}∙k_{w}}{2m}$, which means that with such an expectation of the entire random network its difference is $A_{vw}-\frac{k_{v}∙k_{w}}{2m}$. Thus, the metric of modularity is defined as:

$$Q=\frac{1}{2m}\sum_{vw}(A_{vw}-\frac{k_{v}∙k_{w}}{2m})\delta_{vw}$$ (3)

### 2.5 Data

In the proposed work, the pollutant data samples (O_3_, PM_2.5_, and SO_2_) were collected in the Municipal Monitoring Center (Tianjin City) with a time span of 1 hour for each measurement. As a representative location for data collection, Tianjin city is on the east coast of the mid-latitude Eurasia continent, which is dominated by monsoon circulation. This place is the prevailing area of East Asian monsoon and has a temperate monsoon climate. The main climatic features are four distinct seasons: windy spring (drought and little rain), hot summer (concentrated rain), cool autumn (warm and cool), and cold winter (dry with little snow). Tianjin has a serious air pollution problem mainly caused by inhalable particulate matter, and great efforts have been taken to control the air quality in recent years.

**Fig 2:**
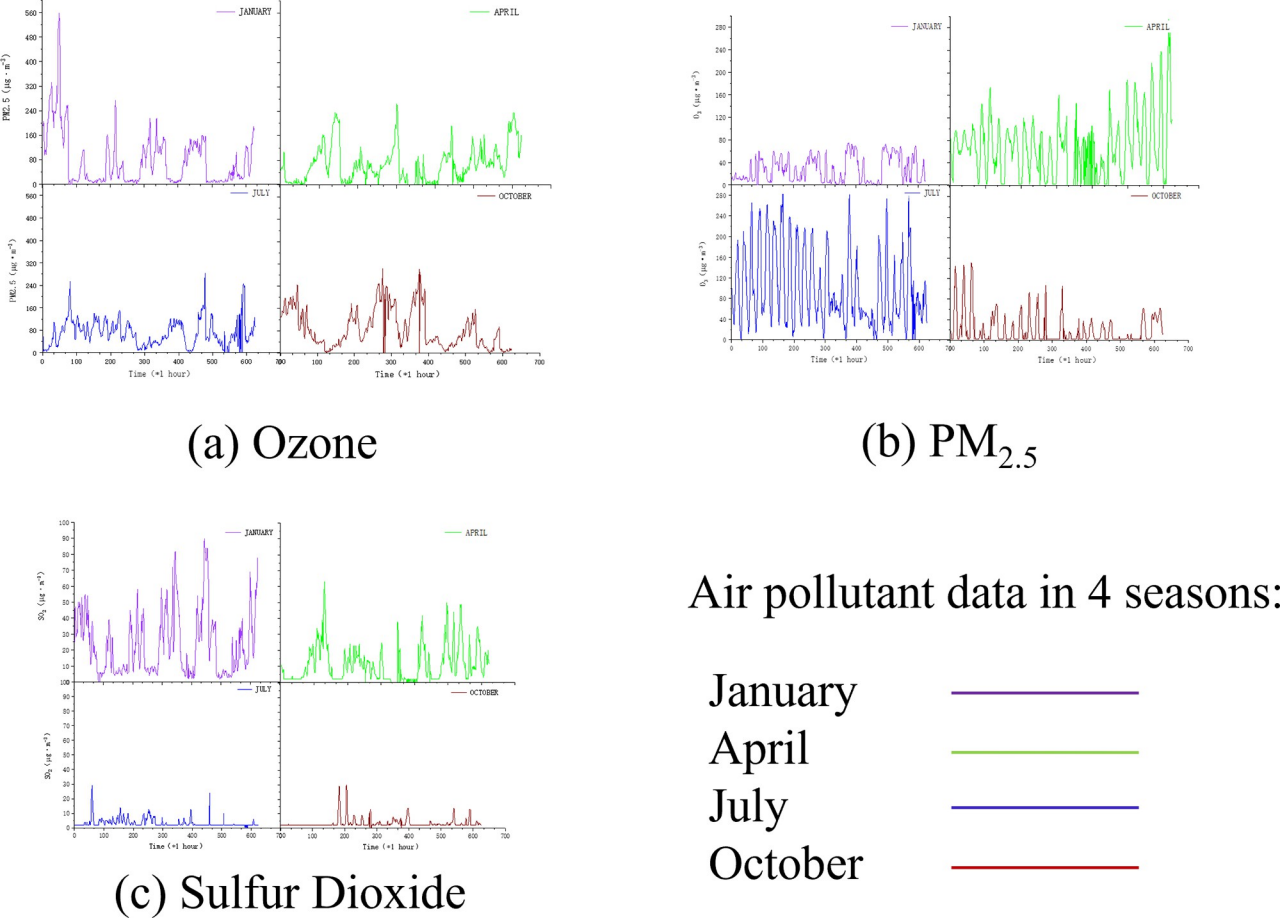
The data of three indicators of pollutants in Tianjin (2016) in different months of four seasons.

Fig 2 illustrates the changes of three pollutant indicators (O_3_, PM_2.5_ and SO_2_) in January, April, July and October of 2016, respectively. It can be seen that the pollutant data of different seasons have different ranges and changing law. For example, the concentration of O_3_ in April and July was relatively high, and the variation range of O_3_ in July was also relatively large in comparison to that of January and October.

### 2.6 Data transformation and analysis

**Fig 3:**
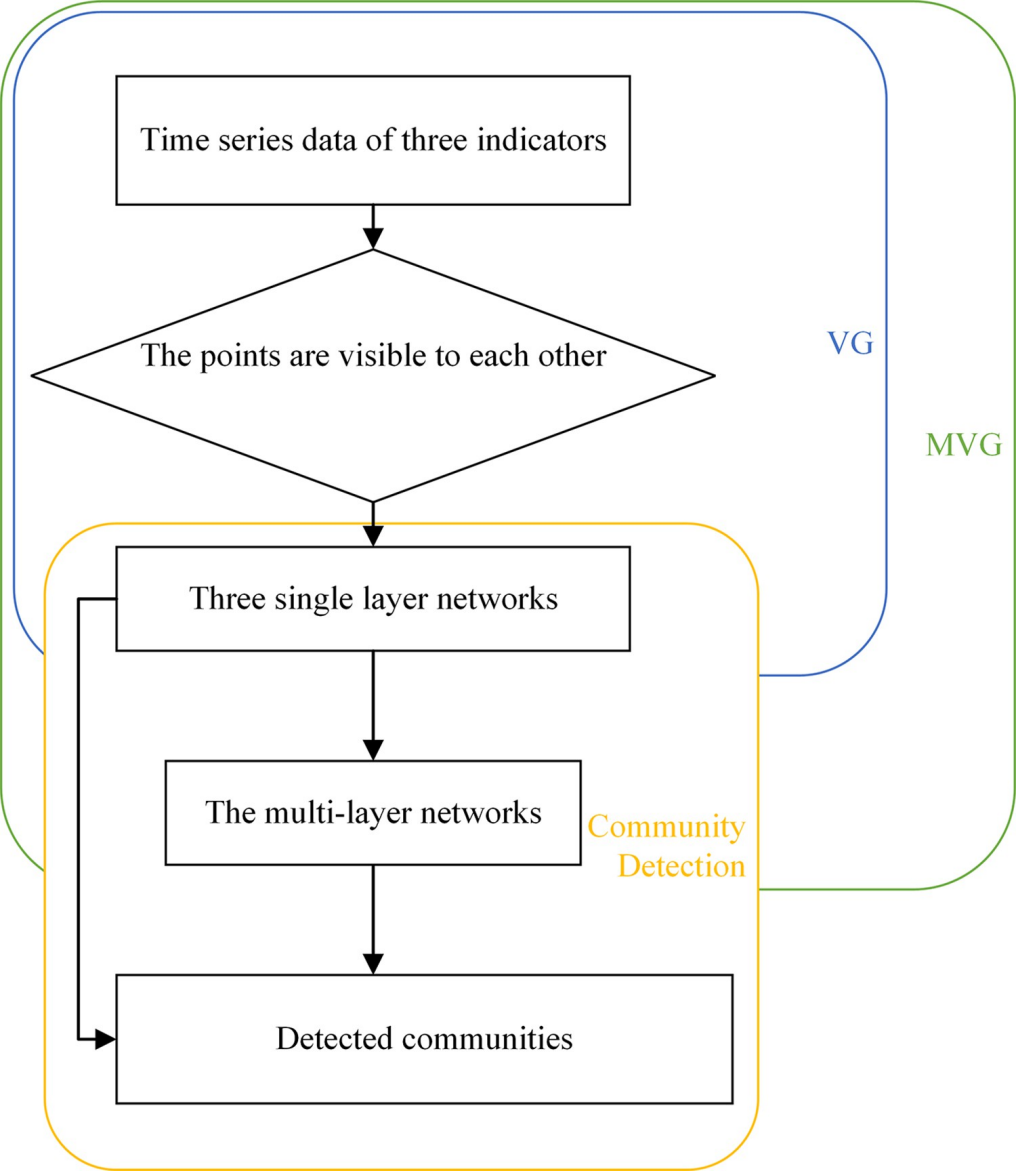
Proposed scheme of network generation and combination.

The scheme of the proposed work is formed by two functional parts as shown in Fig 3. The first part is to build a multi-layer network based on an MVG data transformation (shown as the green color box), and the second part is to perform community detection on the obtained network (shown as the yellow color box).

In Fig 3, the data of O_3_, PM_2.5_ and SO_2_ are firstly transformed into three single-layer complex networks by VG. Then, those independent networks are combined to generate a comprehensive multi-layer network using:

$$r_{ij}=\omega_{a}*a_{ij}+\omega_{b}*b_{ij}+\omega_{c}*c_{ij}$$ (4)

$$\Gamma =\Omega_{a}+\Omega_{b}+\Omega_{c}$$ (5)

where *a*_*ij*_, *b*_*ij*_, *c*_*ij*_ and *r*_*ij*_ denote the element entry (*i*,*j*) in different matrixes of three single-layer networks and the combined multi-layer network, respectively. Let *ω*_*a*_, *ω*_*b*_ and *ω*_*c*_ be the weights, the multi-layer network Γ is defined as the weighted product of three matrices from the other single-layer networks. For community identification, the community detection is performed on all the networks to form the nodes in the final network.

**Fig 4:**
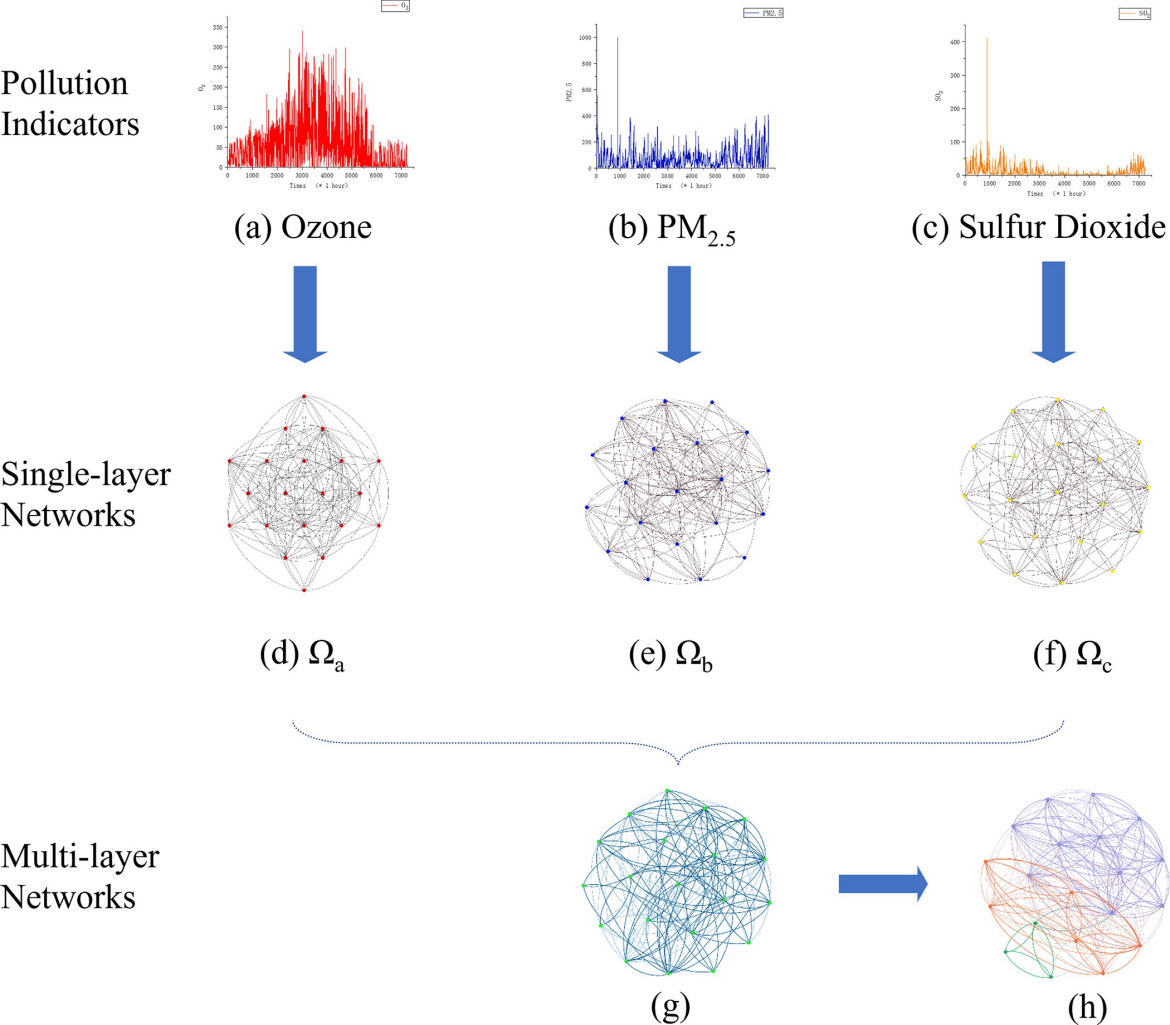
The procedure for constructing the multi-layer network based on the data of time series and community detection. Three single-layer networks (d,e,f) are gotten from time series data of O_3_, PM_2.5_ and SO_2_ (left to right). And the multi-layer network (g) is obtained from the three single-layer networks, then we run the community detection algorithm for this network, turning out the last graph(h).

Fig 4 presents the graphs of data transformation in different stages. Fig 4(A)–4(C) are the data graphs of three indicators: O_3_, PM_2.5_, and SO_2_ collected in 2016. Fig 4(D)–4(F) are three single-layer network graphs generated by 20-time points of three indicators with VG. Fig 4(G) is the combined graph of (d)-(f) using Eq (5) of MVG, which employs the weight of the edge to represent the number of connections between two points in (d)-(f). By taking graph (g) as input, the result of community detection is distinguished in (h) by colors.

### 2.7 Community detection

To detect the communities, each node of the network needs to be initialized into its own community in the beginning, then each pair of the modularity value belonging to the communities (shown as the connected nodes in Fig 4 need to be computed. For the nodes having the highest increasing modularity, they will be moved to the same community. Both modularity computation and node migration are performed alternatively in a loop until no such community pairs that can increase modularity exist any longer. The overall scheme of the proposed work is shown below, and more details can be found in [27].

**Proposed Algorithm of Network Construction and Communication Detection**

**Input:** air data of different indicators $D=\{D_{1},D_{2},\ldots D_{n}\},D_{i}=\{{data}_{t_{1}},{data}_{t_{2}},\ldots {data}_{t_{m}}\}$, the weight coefficient $\omega =\{\omega_{a},\omega_{b},\ldots \omega_{n}\}$

**Output:** collection of communities $C=\{C_{1},C_{2},\ldots C_{k}\},C_{i}=\{t_{1},t_{2},\ldots t_{n}\}$

Obtain single layer networks $N^{1}=\{N_{1}^{1},N_{2}^{1},\ldots N_{n}^{1}\}$ from D by VG:

**For** every 2 nodes *i*,*j* in $N_{x}^{1}$ **do**

**If** any *t*∈(*t*_*i*_, *t*_*j*_): ${data}_{t}<{data}_{t_{i}}+({data}_{t_{j}}-{data}_{t_{i}})\frac{t-t_{i}}{t_{i}-t_{j}}$, **do**

The element in *N*^1^ matrix *a*_*ij*_ = 1

**End**

**End**

Obtain multi-layer networks $N^{2}=\{N_{1}^{2},N_{2}^{2},\ldots N_{n}^{2}\},N^{3}=\{N_{1}^{3},N_{2}^{3},\ldots N_{n}^{3}\}\ldots$ with *ω* by MVG:

$N_{i}^{2}=\omega N_{r}^{1}+\omega_{s}N_{s}^{1}$,

$N_{i}^{t}=\sum_{k=1}^{t}\omega_{r_{k}}N_{r_{k}}^{1}r_{k}\in (1,n)\;and\;r_{i}\neq r_{j}$

**For** *N* in {*N*^1^, *N*^2^, *N*^3^} **do**

Get *P*(*k*) and *γ* of *N*, *P*(*k*)∝*k*^−*γ*^

Each node *n*_*i*_ in *N* belongs to its own community *C*_*i*_

**While** the modularity value *Q* of *C* is not maximum **do**

**For** *C*_*i*_ in *C* **do**

Count the number of edges *ne*_*i*_ in one community *C*_*i*_

Get the edge fraction of *C*_*i*_: *f*_*i*_ = *ne*_*i*_/*ne*, *ne* is the number of edges in N

Compute the expected edge fraction $\tilde{f_{i}}$ by randomly distributings edges in *C*_*i*_.

Get the modularity of *C*_*i*_: $Q_{i}=f_{i}-\tilde{f_{i}}$

**End**

**For** *C*_*i*_, *C*_*j*_ in *C*

Suppose *C*_*i*_ = *C*_*i*_∪*C*_*j*_, compute the modularity change of $C,Q_{ij}^{\prime }$

**End**

**If** $Q_{vw}^{\prime }$ is maximum among $Q_{ij}^{\prime }$ then

*C*_*i*_ = *C*_*i*_∪*C*_*j*_(*i*<*j*)

**End**

**End**

**End**

## 3. Results and discussion

### 3.1 Centrality parameter of networks

**Fig 5:**
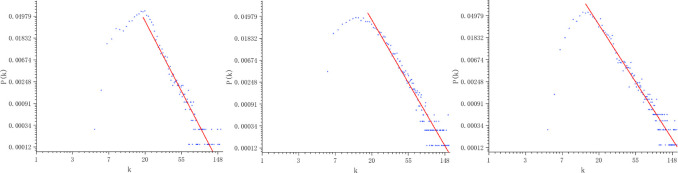
Degree distributions of the three indicators’ network obtained from the data (From left to right: the graph of O_3_, PM_2.5_, SO_2_.).

**Fig 6:**
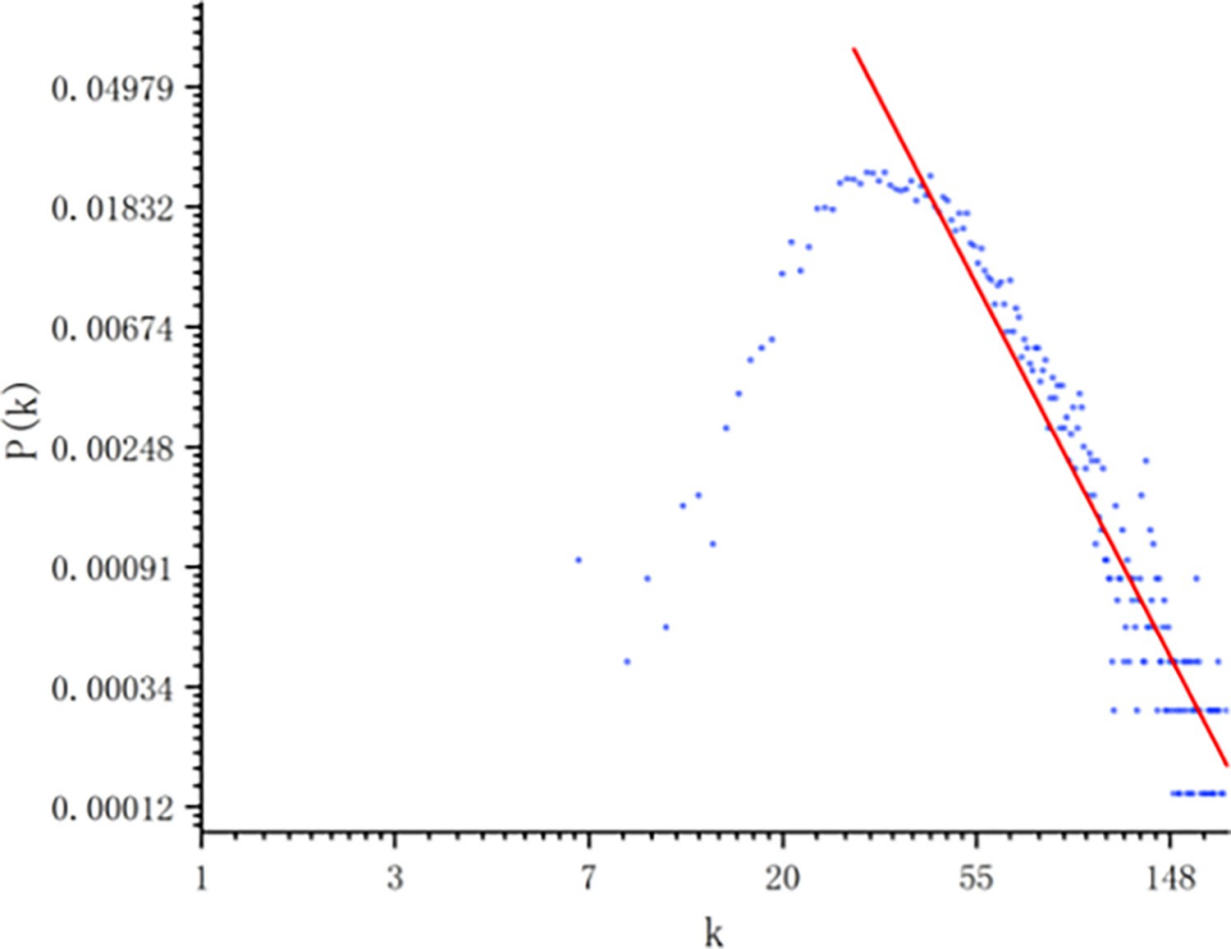
Degree distributions of the multi-layer network obtained from the data.

According to the role of the centrality parameters in [14], the degree distribution is calculated for the networks generated from the data of three indicators, as shown in Fig 5. In Fig 6, the graph of the multi-layer is presented. We notice that all degree distributions of the complex networks show a power-law behavior in the tail of the degree distribution, which indicates a fractal behavior of the time series. From the linear regression in the log-log plot of this tail, γ coefficient is able to be obtained at 3.09759 ± 0.07221.

### 3.2 Community detection

**Fig 7:**
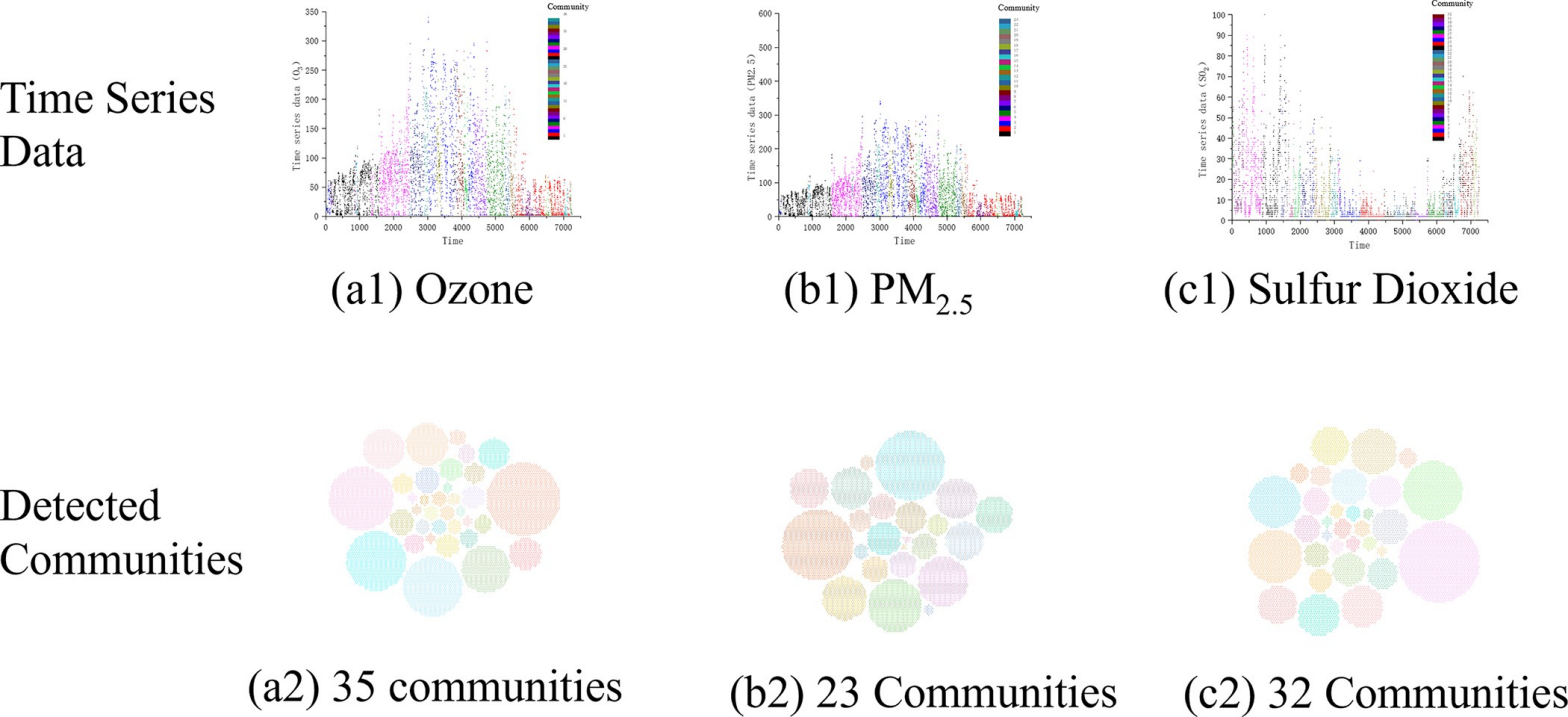
The time series data (1^st^ row) and the detected communities (2^nd^ row) of three representative pollution indicators: (a) O_3_, (b) PM_2.5_ and (c)SO_2_. Each community is annotated with different colors.

To evaluate the community detection on multi-layer networks, an ablation study based on different combination strategies of the single-layer networks is employed for valuation, as shown in Fig 7 and Table 1.

**Table 1:** Ablation study of community detection based on different network combinations.

Indicators for Networks Combination	Detected Community Count		Nodes in the Different Community	Nodes Proportion in the Different Community
**PM**_**2.5**_ **&SO**_**2**_	**8**	1	2167	29.9020%
		2	1682	23.2096%
		3	1042	14.3784%
		4	912	12.5845%
		5	893	12.3223%
		6	534	7.3686%
		7	12	0.1656%
		8	5	0.0690%
**O**_**3**_ **& SO**_**2**_	**11**	1	1669	23.0302%
		2	1470	20.2843%
		3	905	12.4879%
		4	873	12.0464%
		5	867	11.9636%
		6	404	5.5747%
		7	353	4.8710%
		8	331	4.5674%
		9	211	2.9115%
		10	152	2.0974%
		11	12	0.1656%
**O**_**3**_ **& PM**_**2.5**_	**6**	1	1780	24.5619%
		2	1650	22.7680%
		3	1486	20.5050%
		4	1404	19.3735%
		5	857	11.8256%
		6	70	0.9659%
**O**_**3**_ **& PM**_**2.5**_ **&SO**_**2**_	**6**	1	2314	12.7563%
		2	3638	20.0551%
		3	4314	23.7817%
		4	3516	19.3826%
		5	2120	11.6869%
		6	2238	12.3374%

**Fig 8:**
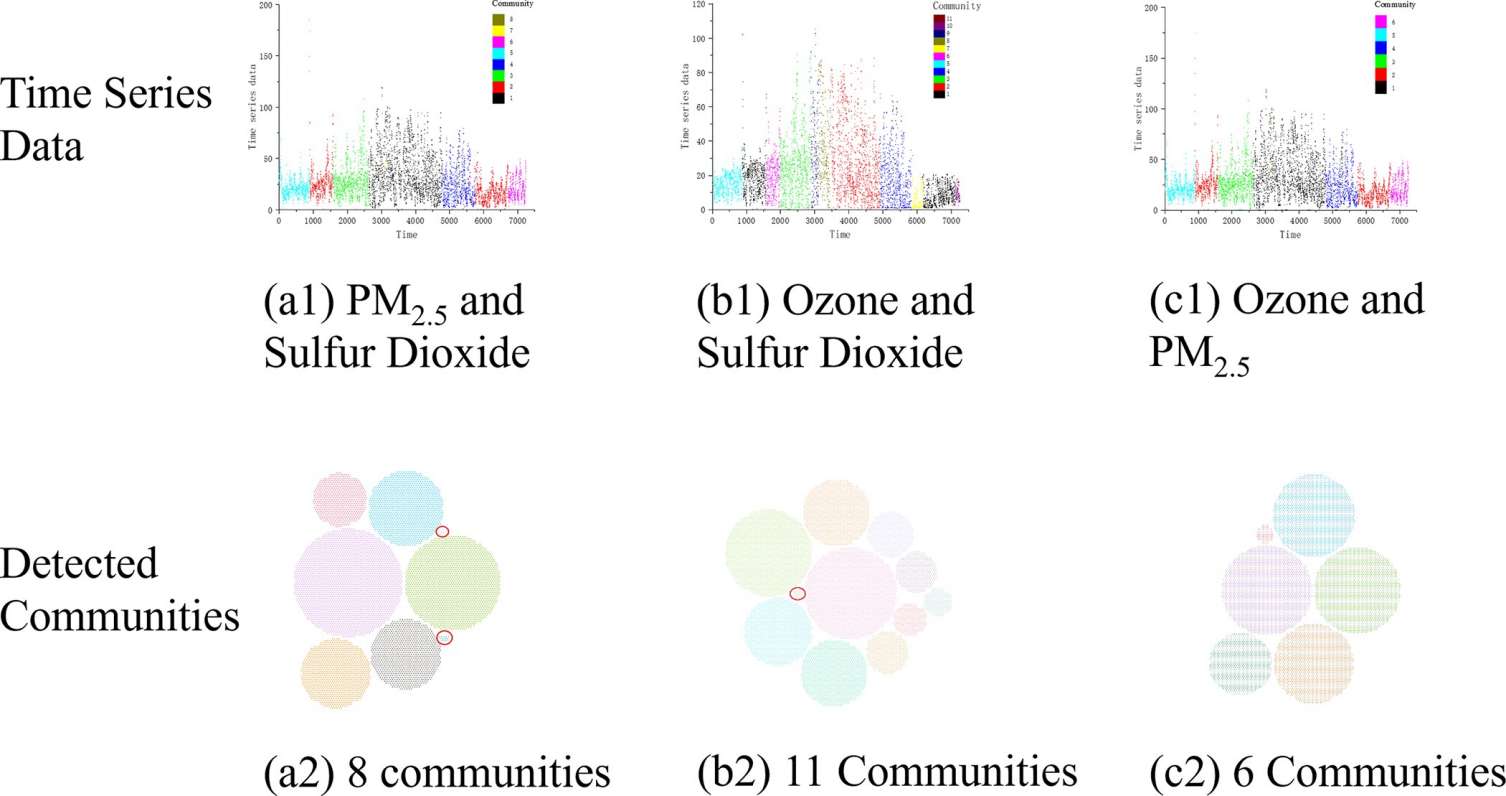
The time series data (1^st^ row) and the detected communities (2^nd^ row) in different multi-layer networks combined by two single-layer networks. Each community is annotated with different colors.

For the pollution indicators **PM**_**2.5**_ **& SO2**, 8 communities are detected in the multi-layer network combined by 2 single-layer networks that is shown in Fig 8(A2). The largest community consists of 2167 nodes, accounting for 29.9% of all the nodes. Two tiny communities of No.7 and No.8, which are marked out by the red circle in Fig 8(A2), contain 12 time points and 5 time points, respectively. They are corresponding to the time periods of 2016-11-3 [23:00]~2016-11-4[10:00] and 2016-06-12[23:00]~2016-06-13[03:00], which are in proportion to 0.0690% and 0.1656% of all the time periods. In comparison to the community of No.1, communities of No.7 and No.8 are not divided properly. From the viewpoint of climate, Tianjin was in the middle of summer at that time, which means that these three communities should only be divided into one community.

For the pollution indicators **O**_**3**_ **& SO2**, 12 communities can be detected in the multi-layer network combined by 2 single-layer networks as shown in Fig 8(B2). There also exists the smallest detected community No.11 (red circle), which contains 12 time points corresponding to the first half of January 1, 2016. But according to the climate of Tianjin at that time, community No.11 should be with Communities No.5 and No.10 in one community because of the same winter time in these periods.

For the pollution indicators **O**_**3**_ **& PM**_**2.5**_, 6 communities can be detected in the multi-layer network combined by 2 single-layer networks, which is as shown as the Fig 8(C2).

**Fig 9:**
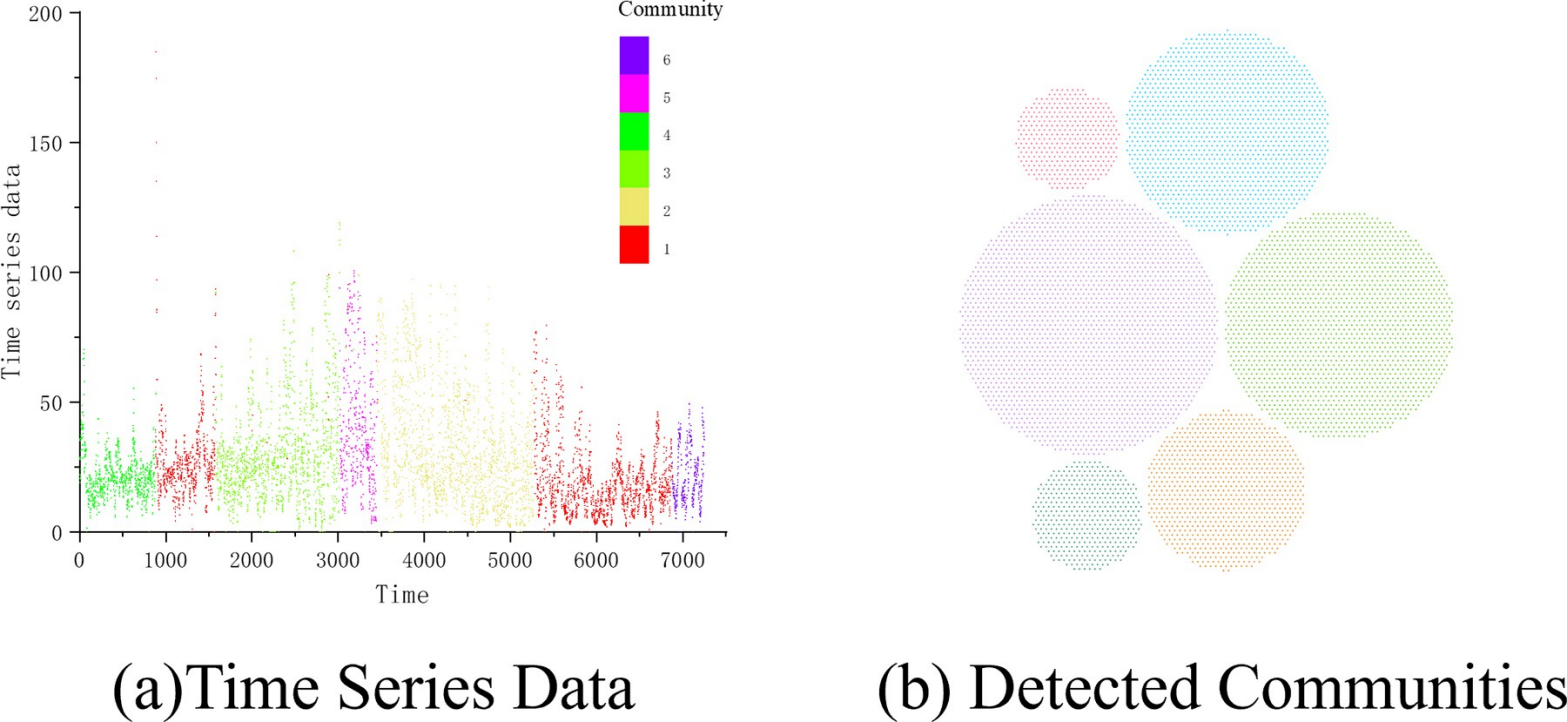
The time series data (a) and the detected communities (b) in the multi-layer network combined by three single-layer networks. Each community is annotated with different colors.

For the pollution indicators **O**_**3**_ **& PM**_**2.5**_**&SO**_**2**_, 6 communities can be detected in the multi-layer network combined by 3 single-layer networks. As shown in Fig 9, the whole year of 2016 is divided into seven periods and distributed into 6 different communities. The biggest community is corresponding to the beginning of February to the middle of March and the middle of September to the beginning of December, which are the periods of late winter and whole summer (from late summer to early winter) in Tianjin. In the year of 2016, the summer started from May 16^th^ to September 27^th^, and the winter started from January 1^st^ to March 15^th^ and from October 31^st^ to December 31^st^, which means that the seasons of spring and autumn lasted only two months in this city. In Fig 9(B), Community No.2 runs from June 20^th^ to September 15^th^, which spans the middle of summer. The period of community No. 5 before No.2 is from May 30^th^ to June 18^th^, while the period of community No. 3 is from March 16^th^ to May 30^th^, which is in spring and early summer. As for the first and last two periods in the figure, community No.4 and Community No.6 are corresponding to the periods from January 1^st^ to February 8^th^ and from December 8^th^ to December 31^st^, which are both belonging to the wintertime.

In fact, the primary sources of PM_2.5_ in Tianjin are the emissions exhausted by diesel vehicles and the coal-burning for heating in winter. During this period, coal-burning plays a decisive role in SO_2_ concentration level [28] as well. All three representative indicators in the experiments have shown a season-related property that is affected by meteorological conditions. This does also mean that dust, fog and other types of weather would also increase the pollutant concentration sharply. Additionally, when the exhaust emissions and coal combustion are not considered as variables, the detected communities are still able to show the seasonality of the pollutant indicators. It demonstrates that the impact of local seasonal changes on the pollutant concentrations as well.

The experiment results have shown that the number of communities detected by the multi-layer network is far less than that by the single-layer networks. Since the same modularity maximization-based community detection is performed with each generated network, the optimal results can be guaranteed with the standard of modularity. That is to say, by using the same detection algorithm, the combination of single-layer networks enables the multi-layer network to obtain completely different results, which are more observable and explanatory. Even though two-layer networks have significantly decreased the community number, the size difference between communities is still too large to reflect the information of time series. Furthermore, community detection becomes more distinctly observable and reasonable with the proposed scheme, and this also substantially demonstrates the effectiveness of the utilization of the multiple visibility graph in clustering problems.

## 4. Conclusion

Environmental analysis based on the data correlation is always challenged by parameter diversity. In this paper, a multi-layer visibility graph method of complex network is proposed to perform community detection with modularity maximization. By using the air pollutant data of multiple representative indicators, the proposed method is able to achieve effective division of the stages for a certain period of time. In addition to obtaining reasonable and explanatory results, data transformation of multiple indexes is also performed via mining the community information in multiple indicators data. But the proposed method’s effectiveness in achieving effective division of stages for a certain period of time may be limited to specific environmental contexts or regions and different environmental factors may require specific parameter settings, and the effectiveness of the proposed method might vary across different datasets and indicators. In addition, expanding the application of the proposed method beyond environmental analysis could open up new avenues for research. Investigating its potential in domains such as public health, social network analysis, or urban planning can provide valuable insights and foster interdisciplinary collaborations.

## Supporting information

S1 Data (ZIP)
